# Supplementary material for: Multiferroic Coupling of Ferromagnetic and Ferroelectric Particles through Elastic Polymers
Source: Polymers (Basel). 2021 Dec 31;14(1):153. doi: 10.3390/polym14010153 (PMC8747388; doi:10.3390/polym14010153)
Supplement: Supplementary file 1 [file polymers-14-00153-s001.zip › polymers-1498345-supplementary.pdf]

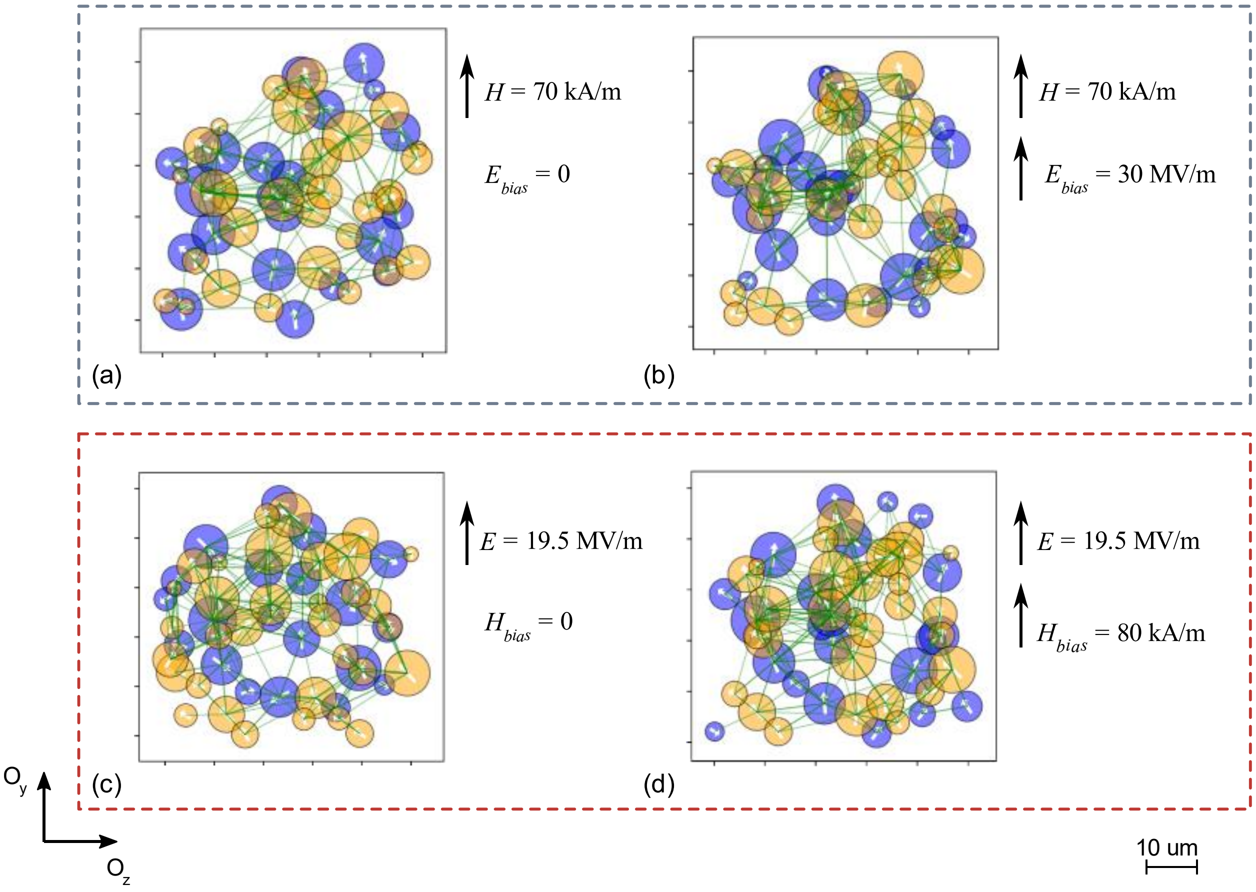

Figure S1. Snapshots of the sample box under the biasing magnetic field H_{bias} = 70 kA/m and acting electric field (a) E = 0 and (b) E = 30 MV/m. Symmetric case: the sample box under under the biasing electric field E_{bias} = 19.5 MV/m and magnetic field of (c) H = 0 and (d) H = 80 kA/m. For clarity of visualization, the polymer beads are made entirely transparent.

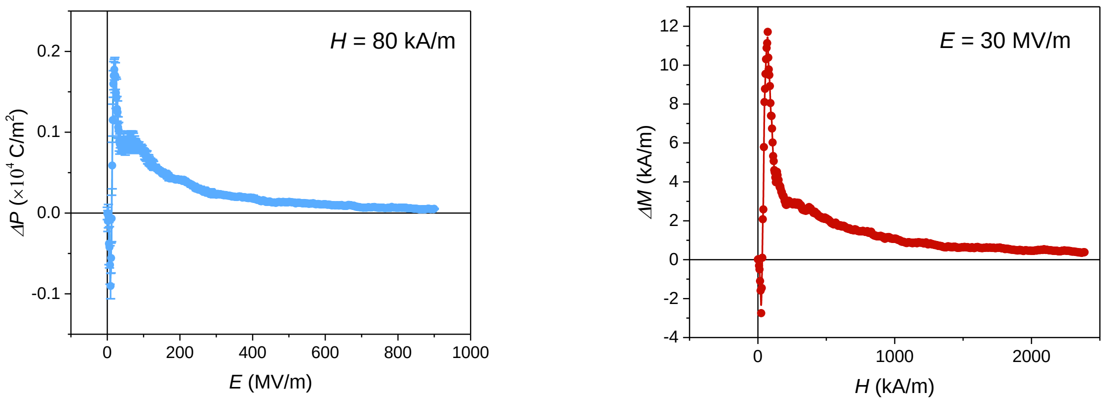

(**a**) (**b**)

Figure S2. Differences between initial and modulated with external field curves: **(a)** polarization change ΔP at the magnetic field 80 kA/m; **(b)** magnetization change ΔM at the electric field strength 30 MV/m.
